# Multiple Transient Idiopathic Small Bowel Intussusceptions in an Adult: A Rare Intraoperative Finding

**DOI:** 10.7759/cureus.110698

**Published:** 2026-06-11

**Authors:** Vinod Ramachandran, Bhovineey Ramanathan, Linda Vu, Patrick Tan

**Affiliations:** 1 Surgery, Royal Perth Hospital, Perth, AUS; 2 General Surgery, Royal Perth Hospital, Perth, AUS

**Keywords:** intussusception, lead point, operation, small bowel, transition point

## Abstract

Adult small bowel intussusception is an uncommon clinical condition and is most frequently associated with a single pathological lead point. The occurrence of multiple synchronous small bowel intussusceptions in the absence of an identifiable cause is exceptionally rare and usually associated with Peutz-Jeghers syndrome. We report the case of a 32-year-old male who presented with persistent lower abdominal pain and was found on imaging to have a short-segment small bowel intussusception without evidence of ischemia. Diagnostic laparoscopy revealed multiple transient entero-enteric intussusceptions occurring at several locations along the small bowel, all of which reduced easily with manipulation; however, they would intussuscept with ongoing peristalsis. No pathological lead point was identified. The patient recovered uneventfully, and follow-up imaging demonstrated no small bowel lesions and no recurrence. This case highlights the diagnostic challenges and management considerations associated with idiopathic, multifocal adult small bowel intussusception.

## Introduction

Intussusception is defined as the telescoping of one segment of the gastrointestinal tract into an adjacent segment, potentially leading to bowel obstruction, compromised perfusion, and ischemia [1,2]. While intussusception is a common cause of abdominal pain in children, it is rare in adults, accounting for approximately 1-5% of intestinal obstructions [3]. Unlike pediatric cases, adult intussusception is typically associated with a well-defined pathological lead point, most commonly neoplastic in nature [2].

Idiopathic intussusception without an identifiable lead point is uncommon in adults, and the presence of multiple simultaneous small bowel intussusceptions is exceedingly rare [4]. With the increasing use of cross-sectional imaging, transient and clinically insignificant intussusceptions are being detected more frequently, complicating clinical decision-making [5]. We present a rare case of multiple transient small bowel intussusceptions identified intraoperatively in an adult patient.

## Case presentation

A 32-year-old male presented to Royal Perth Hospital with a one-month history of lower abdominal pain. The pain was described as a constant pulling sensation with intermittent exacerbations, worsened by sitting upright and relieved by standing or lying flat. He also reported reduced appetite. He developed worsening abdominal pain, nausea, and one episode of vomiting, prompting presentation to the emergency department. His last bowel motion was a day before presentation, and there were no other gastrointestinal or systemic symptoms.

His past medical history was notable as he had recently undergone an abdominoplasty with repair of rectus diastasis and two umbilical hernias four months prior. He was not on any regular medications. He was a current smoker (smoking one cigarette a day) and only consumed alcohol occasionally. There was no history of recent illness or travel.

On examination, the patient was well in appearance with vital signs within normal limits. The blood pressure was 120/60 mmHg, the heart rate was 84 beats per minute, the respiratory rate was 17 breaths per minute, and the oxygen saturation was 96% on room air. Serial abdominal examination revealed a soft but mildly distended abdomen with localized tenderness in the left lower quadrant and associated rebound tenderness. No palpable masses or hernias were identified. The laboratory findings are detailed in Table 1.

**Table 1:** Laboratory investigations.

Parameters	Obtained values	Reference range
Hemoglobin	148 g/L	135–175 g/L
White cell count	12.78 × 10⁹/L	4–11 × 10⁹/L
C-reactive protein	2.2 mg/L	<5 mg/L
Lactate	1.1 mmol/L	<2 mmol/L

**Figure 1:**
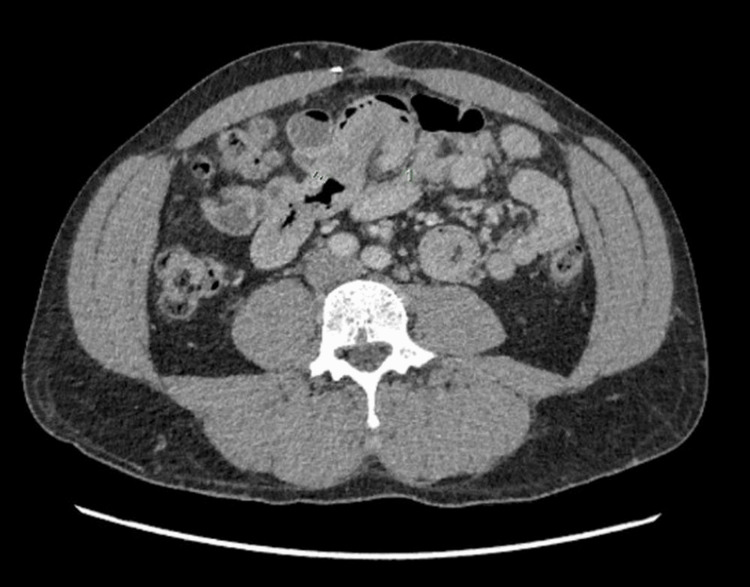
CT scan of the abdomen demonstrating an area of intussusception resulting in a mushroom sign.

**Figure 2:**
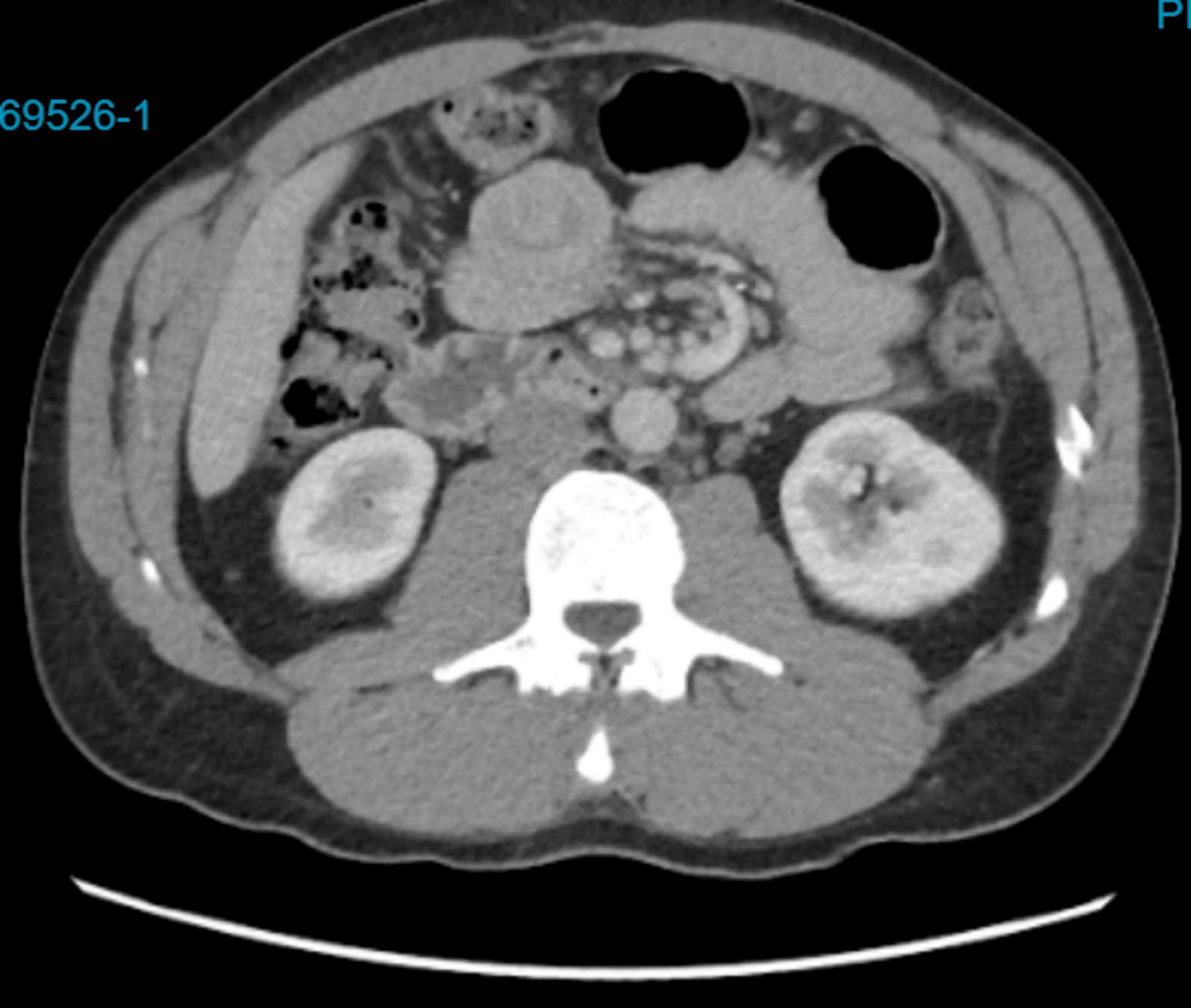
CT scan of the abdomen demonstrating a different area of intussusception with the target sign and proximal thickened jejunal bowel loops.

**Figure 3:**
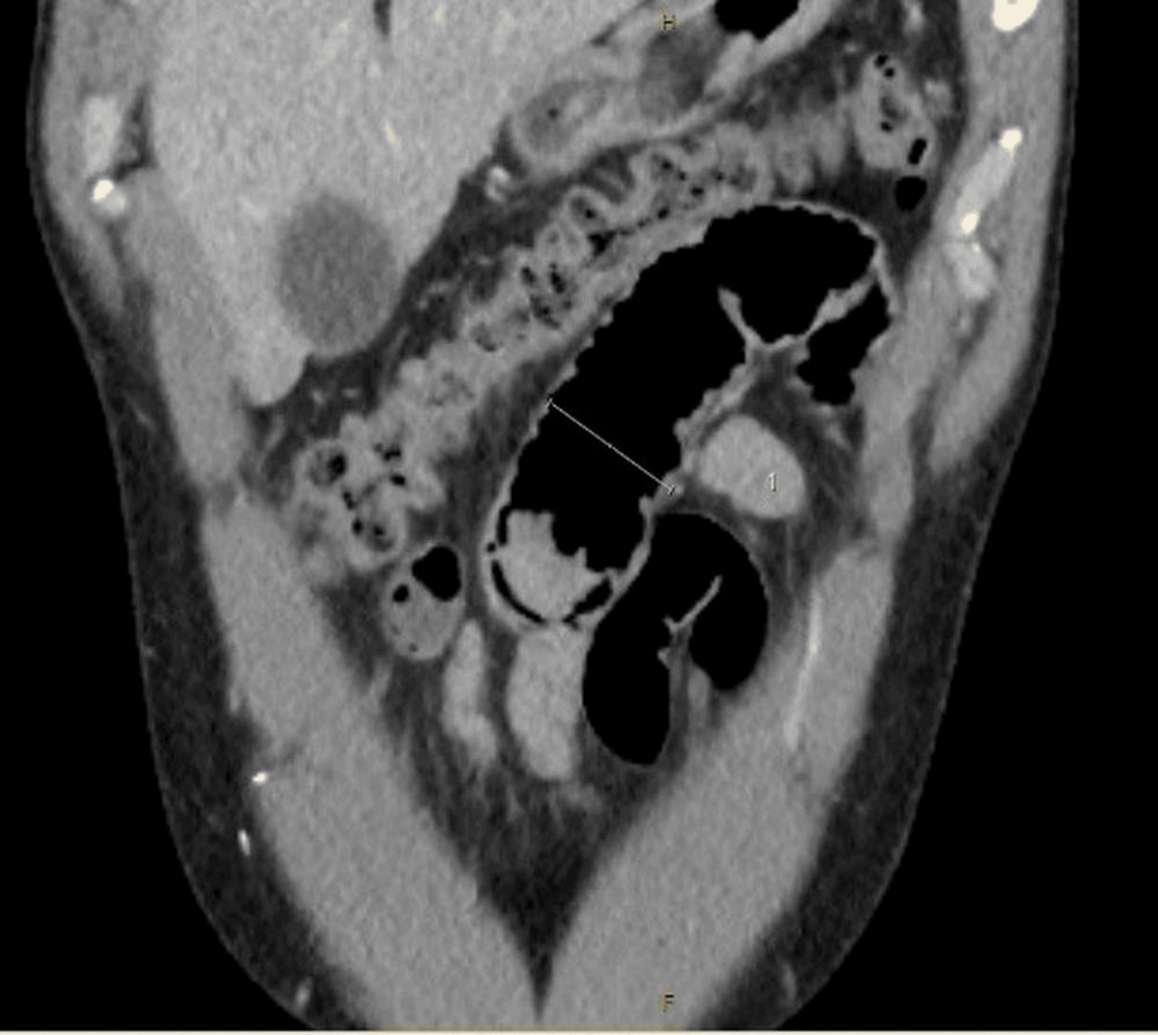
Coronal view demonstrating dilated loops of the small bowel measuring 35 mm.

CT of the abdomen and pelvis revealed a short-segment small bowel-to-small bowel intussusception measuring approximately 3 cm in the right abdomen, with no identifiable lead point. A proximal small bowel loop was mildly dilated to approximately 3 cm. Additional findings included segmental wall thickening involving proximal jejunal loops in the left upper quadrant. There were no radiological features of bowel ischemia. Mild splenomegaly was also noted. The image of the CT scan shown in Figure 1 illustrates a mushroom sign, with the CR scan shown in Figure 2 revealing the target sign. Figure 3 presents the coronal view of the CT of the patient demonstrating the extent of dilatation of the small bowel.

Operative approach and findings

The patient was taken to the theater secondary to the clinical picture and imaging findings. Preoperatively, nasogastric decompression and intravenous fluid hydration were performed. He underwent diagnostic laparoscopy using a 10 mm 30̊® camera and two 5 mm working ports.

Multiple areas of intussusception within the small bowel were discovered; the most proximal was 20 cm from the ligament of Treitz, and the most distal was 60 cm from the terminal ileum. During a systematic small bowel run, several segments were observed to intussuscept dynamically with ongoing peristalsis and to reduce spontaneously or with gentle manipulation. All affected segments were easily decompressed, and the bowel appeared viable throughout, with no evidence of ischemia, obstruction, or intraluminal pathology. There were no pathological mesenteric lymph nodes to suggest inflammation or infection as a cause for mucosal edema to cause dysrhythmic peristalsis.

For intraoperative documentation and future localization, 5-mm Ligaclips were placed at the most proximal intussusception site, with additional clips placed at more distal locations; however, dynamic intussception did not recur at these positions after reduction.

**Figure 4:**
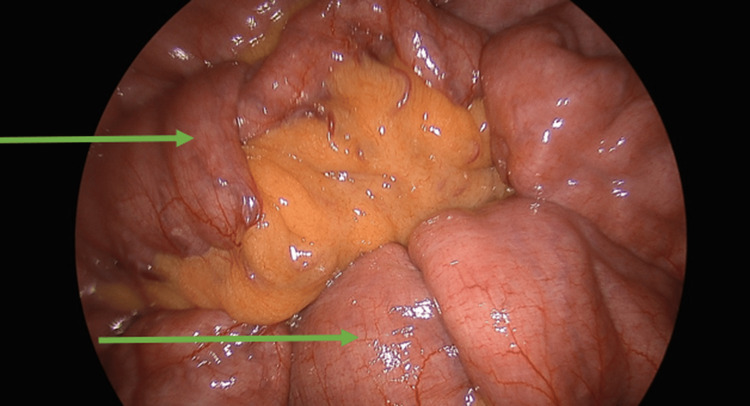
Laparoscopy view with multiple points of intussception demonstrated by green arrows.

**Figure 5:**
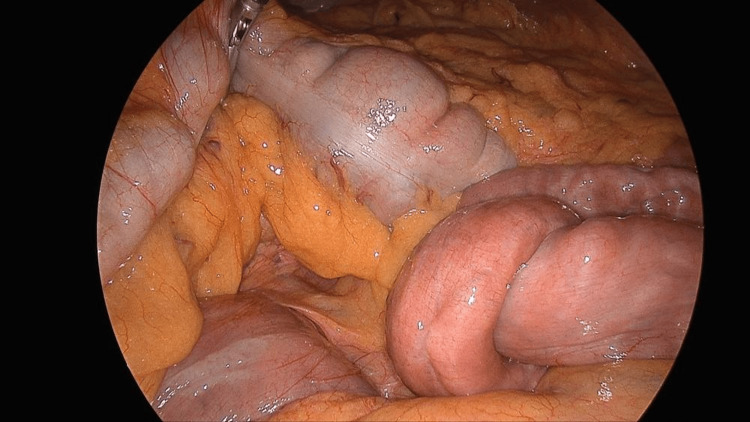
Laparoscopy view showing the most proximal point of intussception by the duodenojejunal flexure.

**Figure 6:**
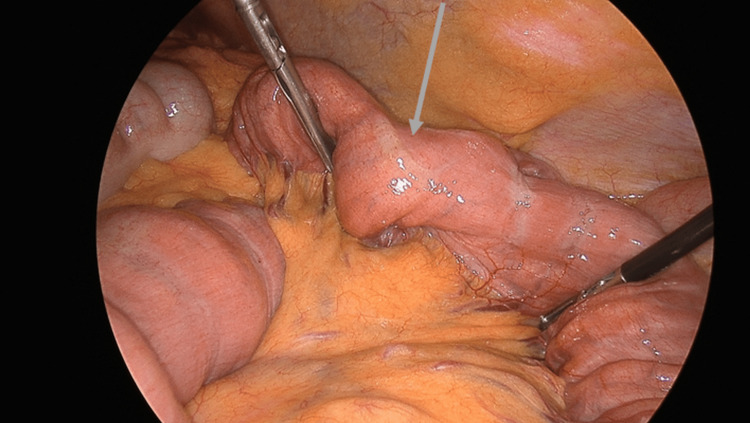
Laparoscopy view. After manipulation of the intussception, white concentric serosal rings are noted (grey arrow).

Figure 4 demonstrates the laparoscopic view with multiple points of intussuception, while Figure 5 reveals the most proximal point of intussuception at the duodenojejunal flexure. Figure 6 reveals the intraoperative image of the bowel after manipulation, noting white concentric serosal rings.

Postoperative recovery and discharge

The patient’s postoperative recovery was uncomplicated. Nasogastric decompression was discontinued on postoperative day three following the return of bowel function, and oral intake was gradually resumed. He remained clinically stable with resolution of abdominal pain and no further gastrointestinal symptoms. The patient was discharged home on postoperative day five with a plan for outpatient magnetic resonance enterography.

Histopathology review

As no bowel resection was performed, no histopathological specimens were obtained for analysis.

Follow-up imaging

**Figure 7:**
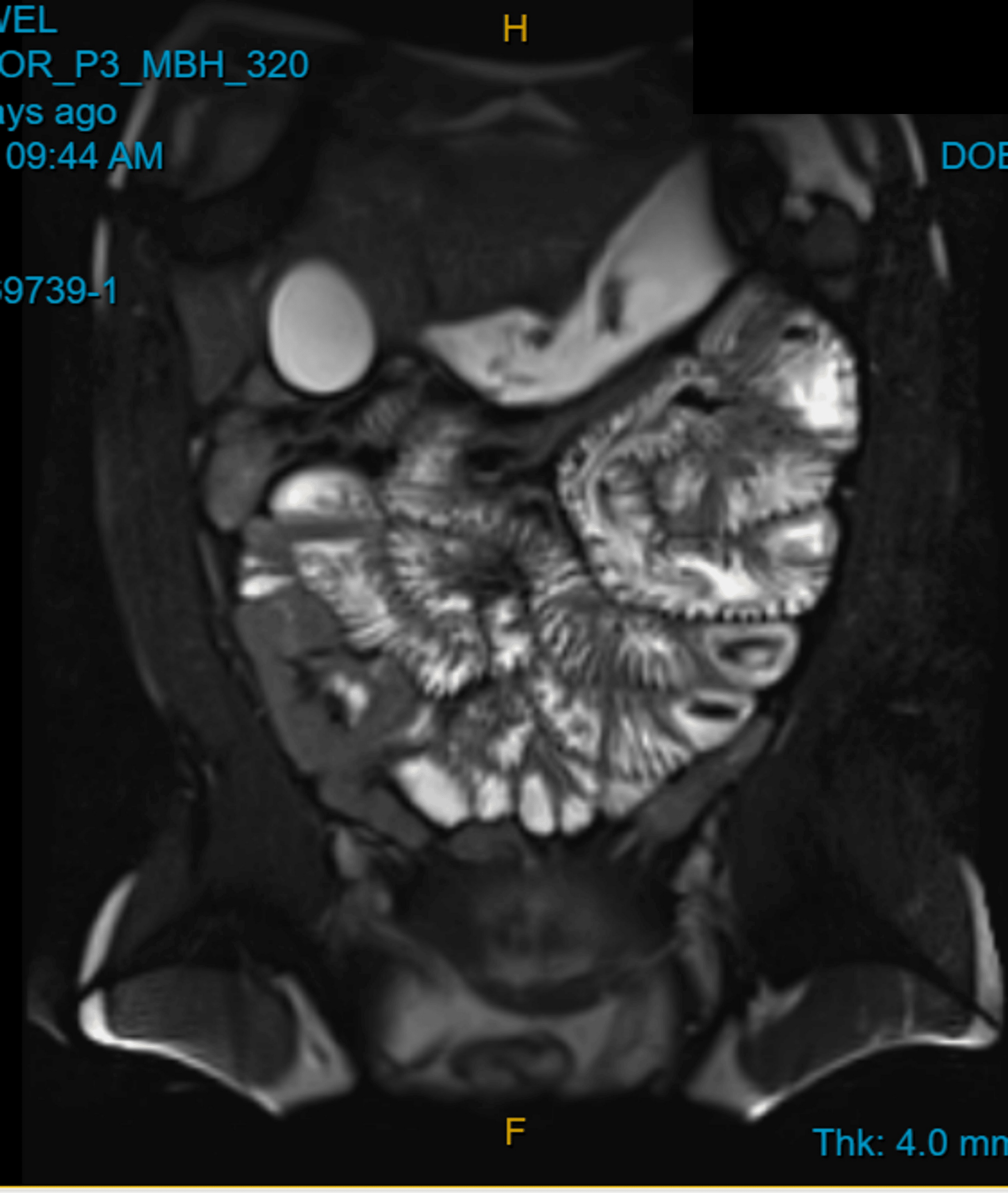
MRI showing no abnormalities.

Magnetic resonance enterography was subsequently performed and demonstrated no abnormalities of the small bowel, with no evidence of recurrent intussusception or an identifiable underlying cause. Figure 7 shows the coronal section of the MRI revealing a normal small bowel appearance with no obvious abnormalities.

## Discussion

Adult intussusception is an uncommon diagnosis and is most often associated with an underlying pathological lead point. Entero-enteric intussusception confined to the small bowel carries a lower risk of malignancy compared with colonic involvement but still warrants careful evaluation [1]. The pathophysiology of adult intussusception remains incompletely understood; however, disruption of coordinated peristalsis due to a focal lesion or altered bowel wall compliance is thought to play a role.

In the absence of an identifiable lead point, proposed mechanisms include transient dysrhythmic contractions, submucosal edema, or functional disturbances in bowel motility. Reports of multiple synchronous small bowel intussusceptions in adults are exceedingly rare [3]. Previously published cases have described similar findings in which multiple short-segment intussusceptions reduced spontaneously or with minimal manipulation, without evidence of ischemia or obstruction, supporting a functional rather than structural etiology [2]. This is the first case to describe ongoing intussception despite manipulation and reduction, likely due to dysrhythmic peristalsis [3].

CT remains the most useful diagnostic modality in adults presenting with non-specific abdominal pain and suspected intussusception [6]. However, with improved imaging resolution, transient and clinically insignificant intussusceptions are increasingly detected, creating uncertainty regarding the need for surgical intervention. While short-segment intussusceptions without obstruction or ischemia may resolve spontaneously, persistent or progressive symptoms often necessitate operative exploration [7].

Surgical management of adult intussusception remains controversial. In idiopathic cases without evidence of ischemia or malignancy, laparoscopic reduction without resection is generally favored to preserve bowel length and minimize morbidity [8]. Conversely, resection without reduction is recommended when a pathological lead point or compromised bowel is suspected [9,10]. In this case, laparoscopy allowed direct visualization of dynamic, transient intussusceptions and facilitated safe reduction without bowel resection.

## Conclusions

This case describes a rare presentation of multiple transient idiopathic small bowel intussusceptions in an adult patient without an identifiable lead point. It highlights the importance of maintaining a broad differential diagnosis in adults presenting with atypical or persistent abdominal pain. Diagnostic laparoscopy plays a crucial role in confirming the diagnosis and guiding management. In carefully selected patients, laparoscopic reduction without resection can be performed safely with excellent clinical outcomes.
